# Stigmatizing Attitudes Toward Persons With Psoriasis in the Western Region of Saudi Arabia

**DOI:** 10.7759/cureus.93946

**Published:** 2025-10-06

**Authors:** Daan T Bagasi, Amal A Kokandi, Lilac R Jamjoom, Refan A Baggazi, Reyof A Jifri, Tala M Maimani

**Affiliations:** 1 Internal Medicine, King Abdulaziz University Faculty of Medicine, Jeddah, SAU; 2 Dermatology, King Abdulaziz University Faculty of Medicine, Jeddah, SAU; 3 Dermatology, King Abdulaziz University Hospital, Jeddah, SAU

**Keywords:** attitudes, emotional rating, psoriasis, social distance, stereotypes, stigma

## Abstract

Background

Psoriasis is a persistent, non-contagious, inflammatory skin illness characterized by remission and aggravation. Further research is needed on the social awareness of psoriasis in Saudi Arabia, particularly in the western region. This study aimed to investigate misconceptions, negative prejudices, and discriminatory behaviors toward individuals with psoriasis.

Material and methods

This cross-sectional study was conducted in four cities (Jeddah, Makkah, Madina, and Taif) in the western region of Saudi Arabia. The participants were ≥18 years of age. Patients with psoriasis were excluded. Data were collected through an online questionnaire distributed through social media applications in July 2023. Frequencies and percentages were used to express all categorical standard deviations, and the mean was used to express quantitative data (mean ± SD). The odds ratios were calculated with 95% confidence intervals (CI). Statistical significance was set at p < 0.05.

Results

This study included 951 participants. Most of the study participants were 20-29 years old (382; 40.2%). A total of 282 (29.7%) participants were men, and 669 (70.3%) were women. The highest frequency of Desire for Social Distance scale items indicated that they would not like to have patients with psoriasis in their homes (51%). Compassion had the highest percentage of endorsement among all emotions on the Emotional Rating Scale (51.4%). On the Myth Endorsement Scale, the most popular response was that psoriasis is caused by poor hygiene (46.1%).

Conclusion

Although most of our population has heard of psoriasis, there is a notable deficiency in the comprehension and awareness of the illness. This highlights the necessity of raising public knowledge about psoriasis, which can be accomplished by educating the public through campaigns, lectures, and seminars at academic institutions, and via brochures and media. Therefore, false attitudes, prejudices, and discriminatory behaviors toward psoriasis could be minimized.

## Introduction

Psoriasis is a persistent, non-contagious, inflammatory skin illness characterized by remission and aggravation. It is an immune-mediated multifactorial condition caused by a combination of immunogenic, genetic, and environmental factors. This is due to increased skin cell proliferation, resulting in increased skin lesions with erythema and silvery scales. The lesions vary in shape and size, ranging from isolated plaques to whole-body involvement. Psoriasis can cause nails to thicken, laminate, and become brittle [1-3].

The prevalence of psoriasis is estimated to be 2-3% worldwide, steadily increasing throughout life. In Saudi Arabia, psoriasis is a prevalent condition, with an estimated prevalence of 5.33% [4]. Despite its high prevalence, there is a lack of awareness and understanding of psoriasis among the general population [5].

In 2014, the World Health Organization (WHO) announced its global aim to reduce stigmatization. WHO member states were urged to implement widespread initiatives to lessen stigmatization. The 2016 WHO global report on psoriasis reaffirmed this demand for action [6]. However, a cross-sectional study conducted in Arabic countries, including 199 Arabic patients with psoriasis, revealed that even satisfied patients with psoriasis suffered from stigmatization. Furthermore, it was observed that the more impaired the quality of life, the higher the level of stigmatization, and the lower the satisfaction with life [7]. A study conducted among non-medical students at Qassim University in Saudi Arabia found that university students required more knowledge of psoriasis [8]. In 2020, another study reported that psoriasis was linked to a significant detrimental impact on patients’ quality of life. Physical deformities and cutaneous lesions might drive patients’ peers to respond negatively, which is the main factor contributing to the psychological burden of illness [9]. Similarly, a study conducted in Riyadh, Saudi Arabia, in 2020 reported that 12.2% of 385 participants considered the disease contagious. Some participants wanted to avoid shaking hands, sharing food, sharing the same swimming pool, and being in a relationship with patients with psoriasis [5]. In 2022, a cross-sectional study in Arar, Saudi Arabia, revealed a positive correlation between psoriasis severity and clinical manifestations and a six-item stigmatization scale due to skin disfigurement. These effects must be addressed in patients to receive better care [10]. Previous studies have shown that social awareness and understanding of psoriasis are crucial for reducing negative attitudes and discriminatory behaviors towards individuals with psoriasis [4,5,7,10]. However, further research is needed on the social awareness of psoriasis in Saudi Arabia, particularly in the western region. While some studies have been conducted in other regions of Saudi Arabia, the western region's cultural differences and unique social norms may impact attitudes and behaviors towards individuals with psoriasis. Therefore, further research is needed to understand this. Few studies have examined how people without medical training perceive psoriasis and react when they see signs of the disease [11].

This study aimed to assess social awareness of psoriasis among the general population in the western region of Saudi Arabia. Specifically, this study investigated the misconceptions, negative prejudices, and discriminatory behaviors toward individuals with psoriasis.

## Materials and methods

This cross-sectional study was conducted in four cities (Jeddah, Makkah, Madina, and Taif) in the western region of Saudi Arabia. The participants were ≥18 years of age. Patients with psoriasis were excluded. All participants were approached using a non-probability convenience sampling technique until an appropriate sample size was obtained. The required sample size was estimated using the Raosoft sample size calculator [12], assuming a population size of 11,000,000, a 5% margin of error, a 95% confidence interval, and a 50% response distribution. The calculated minimum required sample size was 385 [13]. The survey used in this study was freely available, and the original author granted permission for its use and modification upon request and had been previously validated by Pearl et al. [14]. The questionnaire was initially structured in English, translated into Arabic, and back-translated into English to assess the validity of the constructs. After validation, data were collected through an online questionnaire distributed through social media applications in July 2023. Informed consent was obtained from all participants at the beginning of the survey. Ethical approval (reference number 364 23) was received from King Abdulaziz University Hospital's (KAUH) Institutional Review Board in Jeddah, Saudi Arabia. The questionnaire was developed to obtain demographic information regarding the participants’ age, sex, nationality, marital status, region, occupation, and educational level. The survey displayed eight standard images of individuals with psoriasis, with magnified images of psoriatic lesions. Before completing all study measures, the participants completed a manipulation check item to confirm that they had attended to the pictures.

Eight featured images of patients with psoriasis were selected from publicly available internet sites to represent a variety of demographic characteristics and skin lesion locations (e.g., leg, arm, and scalp). Images were shown to the participants in a study first to see how they thought the people in the pictures looked.

This study used a variety of scales that were initially used for research on stigmatized diseases and modified them to study psoriasis. Dermatologists specializing in psoriasis played a crucial role in creating all the items on these scales [15-21].

One of these scales is the Desire for Social Distance Scale, which gauges the degree to which participants wish to distance themselves from individuals shown in the provided images in various social contexts [16]. The scale consists of nine items rated from 1 to 5. The item scores were then averaged, with a higher average score indicating a more pronounced desire for social distancing.

The participants’ emotional reactions to the images were measured using several items [19]. These items asked for ratings of six emotions on a scale of 1 to 5: compassion, pity, disgust, blame, contempt, and curiosity. 

This study also evaluated how the participants endorsed stereotypes about people with psoriasis [18]. This was done using a semantic differential scale composed of 11 pairs of adjectives, including clean and dirty. Participants had to choose one of five circles closest to the adjective that they felt best described someone with psoriasis. The choices were coded from 1 to 5, with 5 being the nearest to the negative adjective. The scores were then averaged, with higher averages indicating a more robust endorsement of negative stereotypes.

In addition, the degree to which the participants endorsed myths about psoriasis was measured [20]. This was done through 15 statements representing common misconceptions regarding the disease. The participants provided ratings to indicate their level of agreement with the given statements using a numerical scale ranging from 1 to 5, where a higher average score indicated a stronger myth endorsement.

Finally, the participants were asked if they had ever been diagnosed with psoriasis, knew someone who had it, or had heard of it, using a Microsoft Excel spreadsheet for data entry and IBM Corp. Released 2020. IBM SPSS Statistics for Windows, Version 26. Armonk, NY: IBM Corp. for data analysis. Frequencies and percentages were used to express all categorical standard deviation (SD), and the mean was used to express quantitative data (mean ± SD). Furthermore, to assess the ratings of social distancing, stereotypes, and myths, the researchers calculated the percentage of individuals who achieved a score of 4 or 5 on each item, indicating a higher level of endorsement. These calculations generated dichotomous variables specific to each item, referred to as "prevalence" variables. Researchers employed a linear regression model to analyze the effects of participants’ age, sex, nationality, marital status, region, occupation, and educational level on all continuous outcomes (social distance, emotional responses, stereotypes, and myths), and whether the participants had heard of, known someone with, or received a diagnosis of psoriasis. The Odds Ratio (OR) was calculated using a 95% Confidence Interval (CI). Statistical significance was set at p < 0.05.

## Results

This study aimed to evaluate the level of social awareness of psoriasis within a general community residing in the western region of Saudi Arabia. The present study examined misconceptions, unfavorable attitudes, and discriminatory behaviors toward patients diagnosed with psoriasis.

This study included 951 participants. Most of the participants in this study were 20-29 years old (382; 40.2%). A total of 282 (29.7%) patients were men, and 669 (70.3%) were women. Saudis accounted for 848 (89.2%) participants and non-Saudis, 103 (10.8%). A total of 458 (48.2%) participants were single. Most respondents were highly educated at the university level or above (75%). Most of the participants were employed (33.5%). Approximately half of the participants reported that they knew someone with psoriasis (427, [44.9%]), and 712 (74.9%) had heard of psoriasis before completing the survey (Table 1).

**Table 1:** Distribution of the participants according to their demographic data, if they know someone with psoriasis and if they had heard of psoriasis before [14]

Variable	No. (%)
Age	
18-19	89 (9.4)
20-29	382 (40.2)
30-39	170 (17.9)
40-49	180 (18.9)
50-59	74 (7.8)
60-69	44 (4.6)
>69	12 (1.3)
Gender	
Female	669 (70.3)
Male	282 (29.7)
Nationality	
Non-Saudi	103 (10.8)
Saudi	848 (89.2)
Marital status	
Divorced	41 (4.3)
Married	434 (45.6)
Single	458 (48.2)
Widowed	18 (1.9)
Educational level	
Illiterate	7 (0.7)
Read and write	6 (0.6)
Primary	4 (0.4)
Middle	16 (1.7)
Secondary	205 (21.6)
University and above	713 (75)
Occupation	
Retired	77 (9.1)
Employed	319 (33.5)
Housewife	138 (14.5)
Unemployed	104 (10.9)
Student	313 (32.9)
Residence	
Jeddah	332 (34.9)
Madina	121 (12.7)
Makkah	277 (29.1)
Taif	221 (23.2)
Do you know anyone with psoriasis?	
No	524 (55.1)
Yes	427 (44.9)
Have you ever heard with psoriasis before completing this survey?	
No	239 (25.1)
Yes	712 (74.9)

The means and SD of the scales used are listed in Table 2. The mean value of the Desire for Social Distance Scale was 2.65 ± 0.93, and the mean total value of the Emotions Ratings scale was 2.09 ± 1.06. The mean Stereotype Endorsement Scale score was 3.04 ± 0.63, and the mean score of the Myth Endorsement Scale was 2.52 ± 1.83.

**Table 2:** Mean and standard deviation of the desire for social distance scale, emotions ratings, stereotype endorsement and myth endorsement [16,18-20]

Variable	Mean ± SD
The Desire for Social Distance Scale	2.65 ± 0.93
Emotions Ratings	2.09 ± 1.06
Stereotype Endorsement	3.04 ± 0.63
Myth Endorsement	2.52 ± 1.83

**Figure 1:**
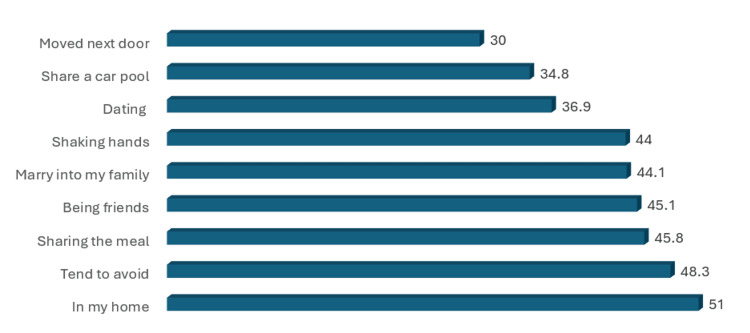
Percentage of endorsement for different desire for social distance scale items [16]

**Figure 2:**
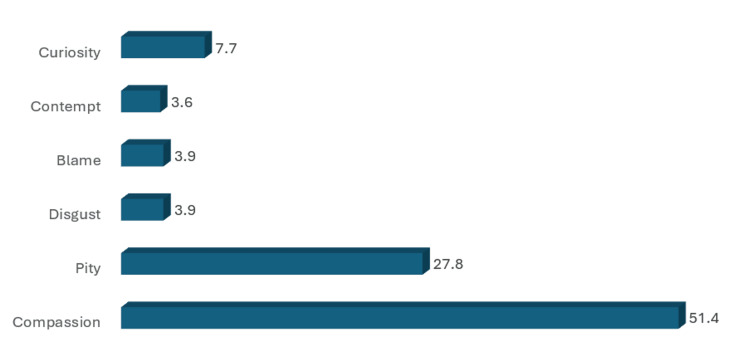
Percentage of endorsement for different emotions ratings scale items [19]

**Figure 3:**
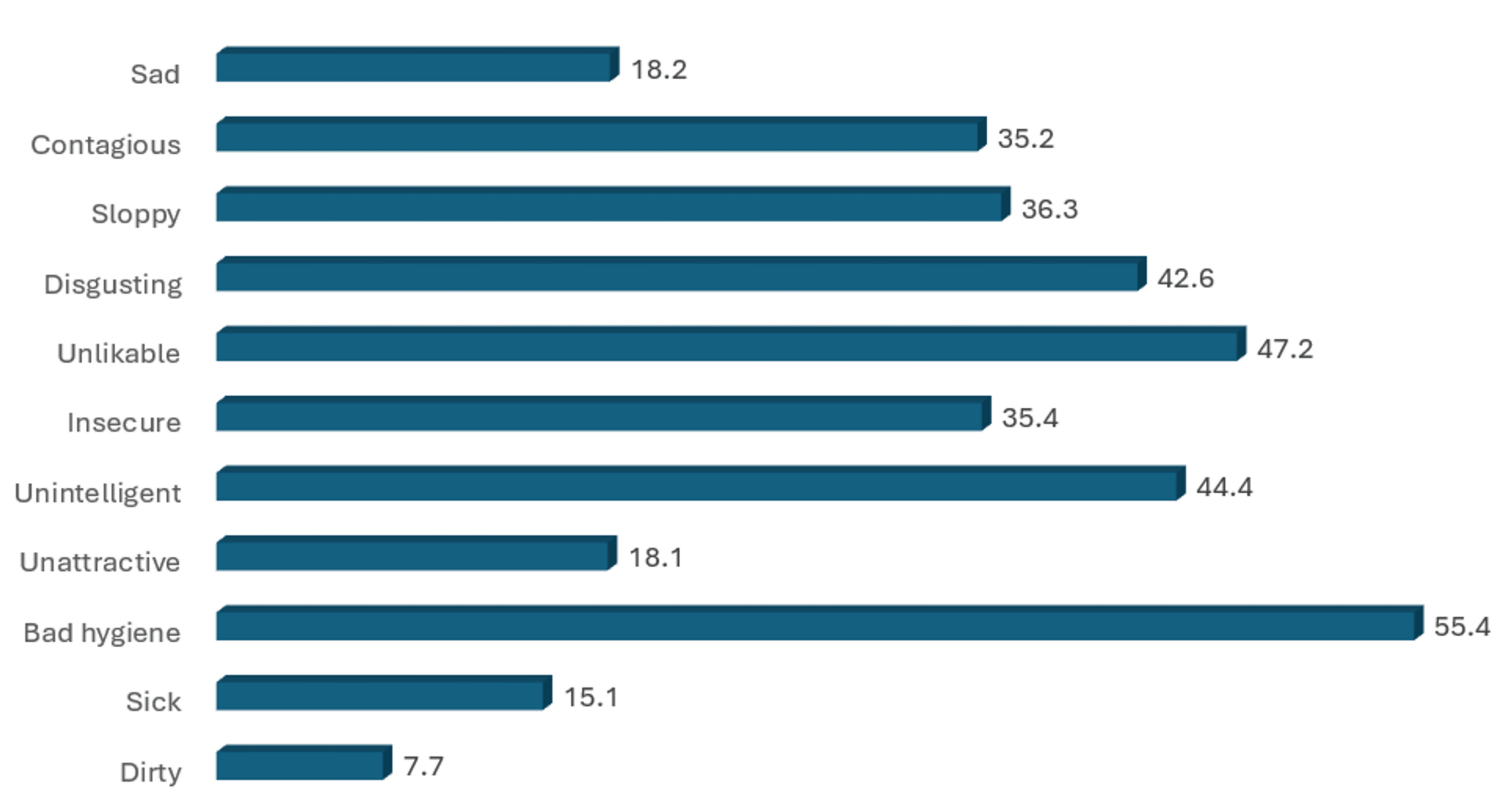
Percentage of endorsing negative stereotypes of persons with psoriasis [18]

**Figure 4:**
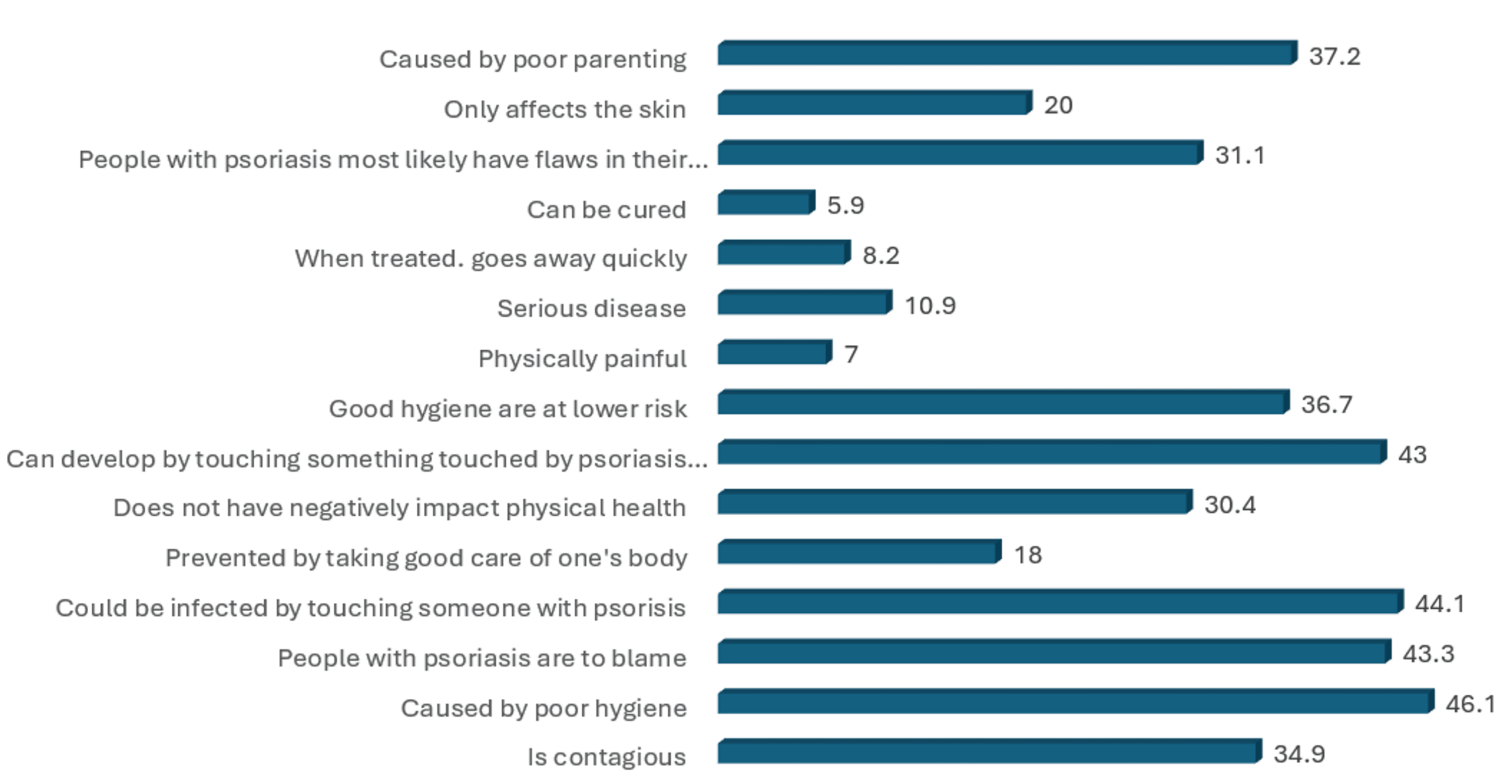
Percentage of endorsing myths about persons with psoriasis. [20]

The prevalence of stigmatizing attitudes is illustrated in Figures 1-4. It was found that the items endorsed with the highest frequency in the desire for Social Distance scale were “have in one's home” (51%), “tend to avoid” (48.3%), ”sharing a meal” (45.8%), “being friends” (45.1%), and “marry into my family” (44.1%) (Figure 1).

Regarding the Emotional Rating Scale, compassion had the highest percentage of endorsement, followed by pity (27.8%) (Figure 2). According to the Stereotype Endorsement Scale items, the items with the highest frequencies were bad hygiene (55.4%), unlikable (47.2%), and unintelligent (44.4%) (Figure 3).

The percentages of myths endorsed by people with psoriasis are illustrated in Figure 4. It was found that the items endorsed with the highest frequency on the myth endorsement scale were that psoriasis is caused by poor hygiene (46.1%), one could be infected by touching someone with psoriasis (44.1%), and that psoriasis can develop by touching something touched by patients with psoriasis (43%).

Multivariate logistic regression analysis was performed to assess the independent predictors of high scale scores, indicating stigmatizing attitudes among the participants. None of the studied variables were predictors of stigmatizing attitudes based on the Desire for Social Distance Scale (p>0.5). Not hearing about psoriasis before completing the survey was a predictor of stigmatizing attitudes according to the Emotions Rating Scale and Stereotype Endorsement Scale (p<0.05) (Table 3).

**Table 3:** Multivariate logistic regression analysis of the independent predictors of high scales score indicating stigmatizing attitudes among studied participants [16,18-20]

	The Desire for Social Distance Scale			
Variable	B	Wald	p-value	Odds Ratio (CI:95%)
Age	1.1	3.44	0.063	0.33 (0.1- 1.06)
Gender	0.1	0.06	0.795	0.73 (0.39-1.34)
Nationality	0.06	1.12	0.683	0.45 (0.33-1.09)
Marital status	0.14	0.26	0.61	0.86 (0.-1.49)
Educational level	0.12	0.83	0.26	0.18 (0.19-1.79)
Occupation	0.07	0.36	0.54	1.08 (0.31-1.4)
Residence	0.31	0.81	0.071	0.32 (0.44-1.56)
Do you know anyone with psoriasis?	0.19	0.49	0.48	0.82 (0.48-1.41)
Have you ever heard with psoriasis before completing this survey?	0.35	1.08	0.299	1.42(0.72-2.79)
	Emotions Ratings			
Variable	B	Wald	p-value	Odds Ratio (CI:95%)
Age	0.31	1.18	0.27	1.36 (0.77-2.4)
Gender	0.03	0.02	0.885	1.03 (0.63-1.68)
Nationality	0.2	0.34	0.556	1.25 (0.59-2.66)
Marital status	0.29	1.73	0.188	0.74 (0.47-1.15)
Educational level	0.04	0.08	0.826	0.95 (0.65-1.4)
Occupation	0.01	0.03	0.858	1.01 (0.82-1.25)
Residence	3	8.38	0.14	0.73 (0.59-0.9)
Do you know anyone with psoriasis?	0.15	0.98	0.075	0.13 (0.14-1.22)
Have you ever heard with psoriasis before completing this survey?	0.32	2.34	0.004	1.2 (1.33-3.5)
	Stereotype Endorsement			
Variable	B	Wald	p-value	Odds Ratio (CI:95%)
Age	0.17	0.83	0.361	0.84 (0.58-1.21)
Gender	0.4	6.31	0.012	1.66 (1.48-3.91)
Nationality	0.09	0.19	0.659	0.9 (0.58-1.4)
Marital status	0.02	0.03	0.85	0.97 (0.73-1.28)
Educational level	0.05	0.09	0.75	1.05 (0.56-1.45)
Occupation	0.08	1.81	0.178	1.09 (0.96-1.24)
Residence	0.1	3.13	0.177	0.88 (0.79-1.01)
Do you know anyone with psoriasis?	0.4	0.9	0.119	0.13 (0.9-1.3)
Have you ever heard with psoriasis before completing this survey?	0.5	7.57	0.006	1.23 (1.4-4.56)
	Myth Endorsement			
Variable	B	Wald	p-value	Odds Ratio (CI:95%)
Age	0.96	0.75	0.384	0.38 (0.04-3.3)
Gender	1.24	3.21	0.073	0.28 (0.07-1.12)
Nationality	0.48	0.29	0.585	1.62 (0.28-2.18)
Marital status	0.74	0.13	0l.87	0.47 (0.08-1.57)
Educational level	0.12	0.93	0.38	0.4 (0.3-1.09)
Occupation	0.17	0.29	0.588	1.19 (0.63-2.23)
Residence	0.18	0.28	0.596	1.2 (0.61-2.36)
Do you know anyone with psoriasis?	0.17	0.3	0.43	0.2 (0.45-1.3)
Have you ever heard with psoriasis before completing this survey?	1.2	0.45	0.199	0.31 (0.48-1.34)

## Discussion

Aim

This study assessed the public awareness of psoriasis in the western region of Saudi Arabia to identify general misconceptions, stereotypes, and discriminatory attitudes towards patients with psoriasis. Emphasis is placed on the resultant strong emotional reactions, endorsement of stereotypes, and the need for educational campaigns to dispel myths and stigmatization.

Emotional rating

In our study, compassion was the most frequently experienced emotion, followed by pity and curiosity. Similar findings were reported in a study conducted in the United States by Pearl et al. [14] and in Riyadh, Jazan, and Germany, where studies recorded very high levels of empathy and pity in responses towards psoriasis patients [5,22,23]. These feelings of true concern reflect the need for more concerted efforts towards attaining awareness and, consequently, understanding and supporting individuals with psoriasis.

Stereotypes and myths

A misconception from the participants was that 55.4% of them found psoriasis to be related to hygiene, and this wrong conception was thought to be culturally influenced by most cultures associating skin diseases with uncleanliness. This finding is supported by related research conducted in Malaysia by Kwan et al. [24], where such a misconception was attributed to a culture that emphasizes hygiene. Studies in the United States and France reported lower frequencies of such misconceptions [14,25]. Furthermore, the persistence of these falsehoods exacerbates the stigmatization suffered by psoriasis patients, making it more challenging for them. According to the findings of Ginsburg et al., this is the reason why up to 19% of patients with psoriasis who portray such behavior have, at one point, been asked to leave a place, for example, a hair salon or swimming pool, because of their condition [26].

One common finding in these studies, which were conducted on different continents and in different countries, is the low level of public awareness about psoriasis. We recommend intensive campaigns and informative social media content to improve the knowledge of the population. Moreover, recent local studies conducted in Qassim, Tabuk, and Jazan [8,9,22] found that respondents mainly received information from family, friends, and social media, with no authentic sources.

Social distancing and stigmatization

Our study found that 51% of the participants preferred not to be in the homes of individuals with psoriasis, and 48.3% avoided those with visible skin disfigurements. Stigmatization and cultural misunderstandings lead to discriminatory practices, as previously reported by Weiss et al. and Chaturvedi et al. [27,28]. Furthermore, psoriasis significantly impacts the quality of life of affected individuals’ families and partners [29]. Such attitudes significantly affected the quality of life of patients with psoriasis and their families. The most unpleasant feature was how others glanced at their skin conditions. The most important features of stigmatization were the fear of rejection and emotions of guilt and shame, the levels of which are substantially connected to pruritus intensity, stress prior to exacerbation, depressive symptoms, and quality of life, as highlighted by Hrehorow et al. [30].

Limitations

This study had several limitations. The cross-sectional design may have introduced recall and information biases. Moreover, restricting the study to the western region limits the generalizability of the findings. To better represent the diversity of the Saudi Arabian population, future studies should adopt simple random sampling and conduct multicenter nationwide surveys. In addition, the reliance on online surveys may exclude populations that do not engage with technology.

## Conclusions

Although most of our population has heard of psoriasis, there is a notable deficiency in the comprehension and awareness of the illness. This highlights the necessity of raising public knowledge about psoriasis, which can be accomplished by educating the public through campaigns, lectures, and seminars at academic institutions, and via brochures and media. Therefore, false attitudes, prejudices, and discriminatory behaviors toward psoriasis could be minimized.
